# The Effect of Vitamins on the Immune Systems of Pigs

**DOI:** 10.3390/ani14142126

**Published:** 2024-07-21

**Authors:** Edda Mainardi, Carlo Corino, Raffaella Rossi

**Affiliations:** Department of Veterinary Medicine and Animal Science, Università Degli Studi di Milano, Via Dell’Università 6, 26900 Lodi, Italy; edda.mainardi@unimi.it (E.M.); carlo.corino@unimi.it (C.C.)

**Keywords:** dietary supplement, nutrition, immune system, pig, vitamin

## Abstract

**Simple Summary:**

Vitamins play a very important role in the immune system as they act as immunomodulators, influencing the host immune response and thereby preventing the development of several diseases. In pigs, there are some physiological phases, including gestation, lactation and post-weaning, during which the animal is exposed to several stressors. Therefore, dietary intervention with doses of vitamins above the dietary requirement could be an effective tool to modulate the immune system, antioxidant status and gut health to prevent disease onset. Given the importance of microelements in the support of the immune system, the present review aims to provide a comprehensive overview of the effects of dietary vitamins on the modulation of immune function and focus on their importance in maintaining pig health.

**Abstract:**

In modern pig farming, there are many environmental, physiological or social stresses that weaken the immune response and increase susceptibility to disease. Nutritional management has a significant impact on the efficiency of the immune system in pigs. Among the various nutrients, vitamins have been shown to have specific effects on immune system activity. However, the needs of modern genetic types are not met by the dietary recommendations for vitamins in pig diets. The present study therefore summarises the data on dietary integration with supranutritional doses of vitamins in gestating and lactating sows and post-weaning piglets in terms of the immune response. The present data highlight that high doses of dietary vitamins are an effective way to improve the immune system, antioxidant status and gut health. Further studies are needed to deepen the understanding of the role of dietary supplementation with vitamins in pigs on immune system and gut functionality.

## 1. Introduction

In modern pig farming, there are many environmental, physiological or social factors that can negatively affect animals’ health and welfare, with an adverse impact on productivity. Among the many factors that can support animal health, the role of nutrition for enhancing the immune system is a key factor that must be considered. High environmental temperatures due to climate change caused by global warming can be a cause of heat stress that negatively affects animals’ productive performances and health and induces changes in barrier function associated with intestinal inflammation in pigs [1]. Furthermore, high stocking densities and confinement can act as a source of social stress, which may result in an increased transmission of pathogens that can impair the immune system and induce immune-related stress [2]. 

Response to stress is linked to a reduced productive efficiency through several factors, including reduced feed intake, increased energy and nutrient expenditure, and increased susceptibility to infections [3]. These factors have the potential to exert a deleterious effect on the immune system, resulting in a diminished immune response and enhanced susceptibility to inflammatory processes that facilitate the colonisation of pathogens. As a result, there has been a decline in productive and reproductive performance, as well as a high incidence of diseases and mortality rate [4]. It is also suggested that the over- and under-responsiveness of the immune system negatively affects pig health and productive performances [5]. So, it is important that animals develop a robust immune response against pathogens, in order to mitigate the adverse effects of pro-inflammatory cytokines [6]. 

A key strategy for the efficient management of pigs is the need to maintain health by reducing the incidence of disease and minimising the use of antimicrobial agents. Among the many factors that can support animal health, the role of nutrition for enhancing the immune system is a key factor that must be considered. In fact, diet composition has a significant impact on the efficiency of the body’s immune defences [7]. There are many nutrients, that have been shown have very important and specific effects on proper immune activity. Given the importance of microelements in the support of the immune system, the present review aims to provide a comprehensive overview of the effects of dietary vitamins on the modulation of immune function and focus on their importance in maintaining pig health.

## 2. Immune System 

### 2.1. Primary and Secondary Lymphoid Organs

Animals have a highly complex and specialised system of organs, with cell types designed to detect and eliminate foreign invaders. They are organised in lymphoid organs and tissues and are responsible for the surveillance, identification, defence and elimination of non-self-agents and are consequently potentially pathogenic. 

The primary lymphoid organs are the thymus and bone marrow, which are involved in the production, differentiation and selection of lymphocytes. The secondary lymphoid organs, involved in antigen uptake and immune activation, are the tonsils (palatine, pharyngeal, lingual), the lymph nodes, the mucosa-associated lymphoid tissue (MALT), which includes the gut-associated lymphoid tissue (GALT), the bronchial-associated lymphoid tissue (BALT) and the spleen [8]. It is also important to consider that GALT represents a significant proportion of the body’s immune cells [9]. It contains up to 70% of the body’s immune cells and is one of the largest lymphoid organs, consisting of both isolated and aggregated lymphoid follicles that constitute Peyer’s patches. Peyer’s patches can be considered the immune sensors of the gut, due to their ability to transport luminal antigens and bacteria. They have different roles, resulting from the complex interaction between immune cells in the lymphoid follicles and the follicle-associated epithelium, such as the induction of immune tolerance or defence against pathogens [10]. In this way, GALT performs several functions, such as enabling the immune system to mount a strong and protective response against pathogens and tolerating feed ingredients and commensal bacteria. A comprehensive review on the anatomical features of the pig immune system has already been published [11]. It is also important to highlight the importance of the pig in biomedical research as an animal model for studying the innate immune system [12].

### 2.2. Innate and Adaptive Immunity

**Figure 1:**
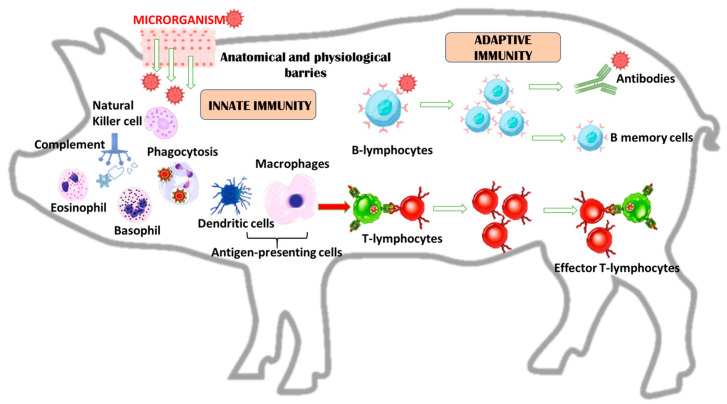
Innate and adaptive immunity in pigs. The figure reports the main cells involved in the immunity response. In the innate response: phagocytes (dendritic cells and eosinophils), granulocytes (eosinophils and basophils) and lymphocytes (natural killer cells). In the adaptive immune response: B-lymphocytes and T-lymphocytes.

The effective action and rapid response of the immune system is essential for good health, and it is mainly mediated by the interaction of the innate and the adaptive immune system as reported in Figure 1. The first line of defence is the innate immune system, which is essential for the maintenance of homeostasis and the prevention of microbial invasion by the elimination of a wide range of pathogens. In fact, it is capable of recognising about ~10,000 different molecular patterns of pathogens [6]. The innate immune system includes physical and chemical barriers and humoral responses and involves many cell types: epithelial cells constitute epithelia, mast cells, dendritic cells, macrophages, monocytes, neutrophils and eosinophils, and natural killer (NK) cells [12]. These components are essential for the control of common bacterial infections. In fact, during primary infection, innate defences can develop an induced response and inflammation [13]. This response is specific due to the cell surface expression of pattern recognition receptors, which can recognise complex polysaccharides, glycolipids, lipoproteins, nucleotides and nucleic acids [14]. 

Adaptive immunity is a more complex, specific and highly effective response against the antigens that trigger it. It is distinguished by its capacity to respond automatically to microbial invasion, to generate a response commensurate with the level of threat, and thus to enhance its efficacy with each subsequent exposure [15]. The adaptive immune response can recognise a pathogen in a specific way and to recognise it on subsequent exposure. It is divided into two main types: humoral immunity and cell-mediated immunity. 

Humoral immunity is an antibody-mediated response that is triggered by the recognition of antigens in the body. These antigens are typically extracellular invaders such as bacteria. This mechanism is primarily driven by B-cell lymphocytes, which produce antibodies after recognising a specific antigen. 

Cell-mediated immunity is primarily mediated by the production of T-cell lymphocytes, macrophages and cytokines in response to an antigen [16]. T cells can be divided into CD8 cytotoxic T cells, which are involved in the direct suppression of damaged or infected cells and tumour cells, and CD4 T helper (Th) cells. The Th cells are important in coordinating the responses of other immune cells, such as B cells, macrophages, and cytotoxic T cells primarily through the secretion of cytokines. T helper cells can be divided into Th1 cells, which produce interferon gamma (IFN-γ) and interleukin (IL)-2 and are important in antiviral and cellular immune responses, and Th2 cells, which produce IL-4, IL-5 and IL-13 and are involved in humoral, antiparasitic and allergic responses. 

In the adaptive immune system, B-lymphocytes are involved in the production of antibodies and immunoglobulin. The B-lymphocyte produces several different structural and functional immunoglobulin (Ig) classes. The different classes of Ig play important roles in defending against pathogens. These functions include pathogen neutralisation, opsonisation for phagocytosis, agglutination, complement activation and antibody-dependent cell-mediated cytotoxicity. In most of these functions, antibodies are also an important link between adaptive specific immunity and innate non-specific immunity.

The activation of the immune response results from the onset of inflammatory phenomena that induce the production of inflammatory cytokines, proteins and enzymes [16]. Cytokines, soluble proteins with low molecular weight, secreted by several immune cell types, are responsible for the dynamic regulation of the maturation, growth and responsiveness of immune cells and are important mediators of the immune system [17]. These proteins can modulate inflammatory processes and restore the physiological equilibrium of the organism. Cytokines can be divided into pro-inflammatory and anti-inflammatory cytokines. Pro-inflammatory cytokines are secreted by T-lymphocytes, macrophages and dendritic cells. They are responsible for the production of many interleukins (ILs), interferon (IFN) γ, β and γ and the tumour necrosis factor α (TNF-α). The main pro-inflammatory cytokines are IL-1, IL-6, IL-8, IL-11, IL-17, IL-18, IL-23, TNF-α, and IFN γ, β and γ, which are responsible for activating the inflammatory response and regulating the host’s defence against pathogens, causing behavioural and physiological changes [18]. Anti-inflammatory cytokines such as IL-10, IL-12, Il-22, IL-37 and IL-38 are secreted by activated macrophages, B- and T-lymphocytes. IL-10 has an immunoregulatory role through the suppression of the immune response, and its main activities are to inhibit the activation of macrophages and the synthesis of pro-inflammatory cytokines [19].

During an acute inflammatory response, cellular and molecular mechanisms are effective in reducing the risk of tissue injury or infection. The resolution of acute inflammation contributes to the restoration of tissue homeostasis. An episode of acute inflammation that is not controlled can, however, become chronic and contribute to a variety of long-term diseases [20]. In an organism in a prolonged inflammatory state, such as chronic inflammation, there is an increased rate of catabolism and cellular metabolism, which leads to the production of free radicals and tissue damage. 

## 3. Nutrition and Immune System

A diet that positively influences immunological outcomes is one that supports the functions of immune cells, enabling them to initiate effective responses against pathogens and rapidly suppress them when necessary. Certain dietary components and micronutrients have been shown to play a specific role in developing and maintaining an effective immune system and/or in reducing chronic inflammation [16]. As previously stated, the production of several components and proteins, including cytokines, occurs when the immune system is activated. In fact, cytokine production activates many defence mechanisms throughout the body. It also activates local cellular responses to infection or injury. To meet the requirements of these processes, the appropriate substrates are required. It is thus evident that a malnourished state leads to a corresponding impairment in the functionality of the immune system. 

The term “malnutrition” is used to describe a deficiency, excess, or imbalance of a wide range of nutrients that has a negative impact on physical condition, physiological function, and clinical outcomes [21]. Malnutrition can be linked to disease-related conditions and can result from several potential causes, including reduced nutrient intake, impaired absorption of macronutrients and/or micronutrients, enhanced losses or altered requirements, and elevated energy consumption in response to diseases [22]. This state is frequently associated with the onset of inflammatory processes, which lead to the onset of enteric disease and a decrease in the gastrointestinal barrier function, resulting in increased morbidity and mortality [16]. Moreover, in such circumstances, a reduction of feed intake was observed due to the production of inflammatory cytokines, with a negative effect on growth performances [23].

The immune system becomes more active when exposed to pathogens, resulting in elevated muscle catabolism and the up-regulation of anabolic pathways for the synthesis of several immune system components [16]. This phenomenon results in an increase in energy, protein, and micronutrient requirements, which elevate the basal energy expenditure. This increased demand for nutrients can be met from exogenous sources such as diet and/or endogenous reserves [24]. The immune system cells utilise glucose, amino acids and fatty acids as energy sources and therefore require various coenzymes, usually derived from vitamins [25]. In addition, some micronutrients and dietary components have very specific roles in the development and maintenance of an effective immune system and in the suppression of chronic inflammatory responses [16]. It is therefore clear that single or multiple nutritional deficiencies often result in compromised immunity. 

**Figure 2:**
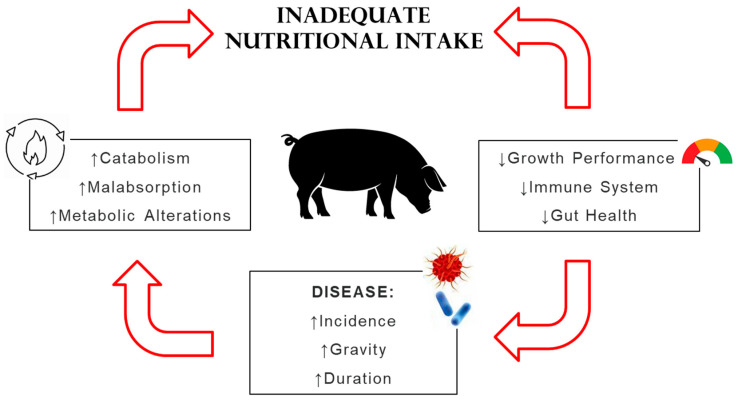
Interactions between malnutrition and inflammation. Inadequate dietary intake can lead to decreased growth performance and impairments in gut health and the immune system that can increase the incidence, duration and gravity of diseases, resulting in metabolic alteration (adapted from Tomkins and Watson, 1989) [26].

It has been demonstrated that pathologies can exacerbate the condition of malnutrition, which further compromises the body’s defences. This evidence shows a clear link between malnutrition and the onset of inflammatory phenomena [25]. These interactions are cyclical and closely connected, and it is appropriate to refer to a cycle linking malnutrition to the onset of inflammatory phenomena [26]. In Figure 2, the cyclical interactions between malnutrition and inflammation are shown.

In consideration of the well-documented relationship between dietary composition and immune function, the concept of immunonutrition has emerged as a field of study that aims to influence the activity of the immune system through targeted nutritional interventions [27]. This approach, firstly applied to human nutrition, was subsequently recognised as a key element in the field of animal nutrition. In fact, in the last few years, the focus in livestock production has shifted to disease prevention, as it has been recognised that improved management and nutrition can reduce mortality and morbidity. Moreover, the dietary integration with specific nutrients and additive may positively modulate the immune system and therefore reduce the therapeutic use of antibiotics [28].

Indeed, in the livestock sector, genetic improvement and modern breeding techniques have led to enhanced productivity and reproductive performance, which resulted in elevated nutrient requirements, particularly during specific physiological phases [29,30].

### 3.1. Micronutrients and Immune System

Micronutrients, such as vitamins and minerals, are key nutrients that are essential for the physiological function of the organism and are supplemented in a small amount [31]. Micronutrients also play a crucial role in the regulation and development of the immune system. Vitamins can influence the correct differentiation of epithelial tissue and support the intestinal barrier by modulating the gap junction, shifting the intestinal microbiota and protecting cell membranes from free radical damage [32,33,34]. Moreover, they also affect immune cells, modulating NK cell function, promoting macrophage activities and boosting cellular immune defences [35,36,37]. Vitamins are also able to modulate the humoral response, modifying antibody production [37,38,39]. Antimicrobial effects have also been reported, modulating the production of interferon γ and the expression of the antimicrobial proteins cathelicidin and defensin [40]. Additionally, it is observed that micronutrients have anti-inflammatory and antioxidant effects [41,42].

Micronutrient deficiencies commonly result in inadequate cellular activity and cytokine secretion, compromising the immune defences. In fact, low levels of vitamins in the diet are directly linked to low levels of NK and phagocytic cell activity and a reduced proliferation of T- and B-lymphocytes [43].

### 3.2. Vitamins and Immune System

Vitamins are a group of organic molecules, precursors of enzymes and coenzymes, that are fundamental for biological processes and are necessary for the proper functioning of all physiological activities in the body. Vitamins are needed in trace amounts in the diet (micrograms to milligrams per day) for health, growth and reproduction [30]. They are divided into two categories according to their solubility: fat-soluble vitamins, including vitamins A, D, E and K, and water-soluble vitamins, including vitamins of the B and C groups. The fat-soluble vitamins also include some precursors of vitamin A, such as carotenoids like beta-carotene. In Table 1, we report the two classes of vitamins with their equivalent names. 

The primary distinction between these two categories of vitamins is their capacity to be stored within the organism. Fat-soluble vitamins are initially digested and absorbed in the intestine, where they are subsequently associated with dietary fats and found in the micelles of the gastrointestinal tract. They are then incorporated into chylomicrons with fatty acids and transported through the lymphatic system into the circulation, where they are stored in the liver and adipose tissue. Three of the four fat-soluble vitamins (vitamins A, D and E) are well stored in significant amounts in the animal’s organism [44]. In contrast to fat-soluble vitamins, water-soluble vitamins are excreted in the urine if consumed in excess amounts (more than the requirement). It is recommended to have a balanced intake of water-soluble vitamins from the B and C groups, except vitamin B_12_, to prevent deficiency symptoms. Water-soluble vitamins are relatively non-toxic, but excessive levels of fat-soluble vitamins A and D can cause adverse effects [45]. The difference in the solubility of vitamins is also important from a nutritional point of view, as it affects metabolism and catabolism. To optimise metabolism, a regular supply of vitamins is required, particularly during the most critical physiological periods when requirements increase. 

**Figure 3:**
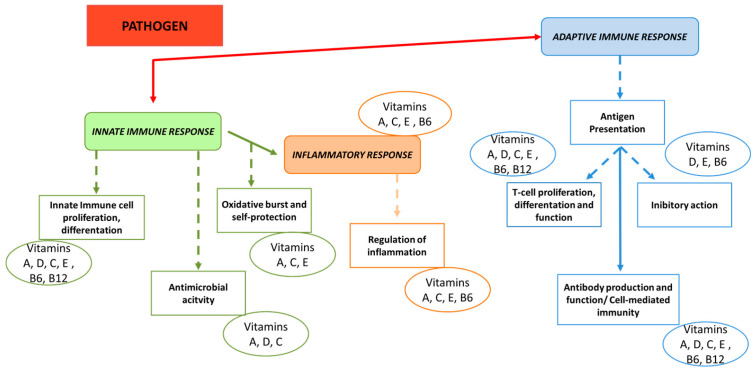
Vitamins involved in innate and adaptive immune responses. This diagram provides a summary of the immune response main processes that are triggered when the body encounters a pathogen, with particular emphasis on the role of vitamins (adapted from Gombart et al.) [40].

Vitamins have a very important and specific effect on the immune system as they act as immunomodulators, influencing the host immune response and thereby preventing and modulating disease development [46]. Dietary vitamin intake may also influence gut health by regulating disease and inflammation, as reported in Figure 3. A deficiency in vitamins increases the risk of intestinal disease; therefore, dietary interventions with doses of vitamins above the dietary requirement could serve as an effective tool to modulate the microbiota and maintain eubiosis, thus preventing the onset of disease [47].

#### 3.2.1. Fat-Soluble Vitamins

Vitamin A can be considered one of the most important vitamins from a nutritional point of view, and it is important as a dietary supplement for livestock. In pig nutrition, synthetic retinyl acetate supplementation is essential to fulfil the dietary requirements of vitamin A, as plant-based feedstuffs are deficient in vitamin A [48]. Vitamin A plays a crucial role in maintaining the functionality of the epithelium of the gastrointestinal tract and has also been shown to promote mucin secretion [49]. Furthermore, it is of great importance for the maturation of the immune system. It influences both cellular and humoral immune responses, promoting the production of secretory IgA and the function of NK cells [50,51].

Vitamin D has long been recognised for its role in maintaining bone health. However, the latest research emphasises that the immunoregulatory function of vitamin D is crucial as its role in bone homeostasis [52]. In pigs, the dietary sources of the active form of vitamin D, 1,25-dihydroxycholecalciferol, are vitamin D_3_ and 25(OH)D_3_. Its role is to mediate both innate and adaptive responses by increasing Ig production and reducing serum concentrations of pro-inflammatory cytokines to maintain immune homeostasis [53]. It is also reported that vitamin D plays an important role in the modulation of intestinal permeability and the production of immunoglobulin A, with an antibacterial function [54,55]. This vitamin is also important in inflammatory conditions, as vitamin D limits cell death in the intestinal tract by promoting the expression of genes for the synthesis of anti-inflammatory peptides such as β-defensin [55].

It is also reported that vitamins A and D have been shown to play a crucial role in maintaining the immune balance at the level of the intestinal mucosa [56].

Vitamin E is not only the most important fat-soluble antioxidant molecule in counteracting oxidative stress but it is also found in high amounts in the cells of the immune system, making it one of the most effective nutrients for modulating immune function [57]. Vitamin E can help maintain membrane integrity, signalling, and the production of key proteins and other mediators, and it directly affects immune cell function by inhibiting lipid peroxidation and associated cell membrane damage [58]. Several studies reported that vitamin E deficiency has been associated to lower feedback of both humoral and cell-mediated immune responses [59]. It is observed, in several animal species, that vitamin E supplementation enhances lymphocyte proliferation, immunoglobulin levels, NK cell activity, and interleukin (IL)-2 production [57]. Moreover, a modulatory activity of vitamin E on pro-inflammatory cytokines such as TNF-α, as well as IL-6 and PGE_2_, was also reported [60].

Vitamin K is recognised as a ‘coagulation vitamin’ due to its ability to contribute to blood coagulation, and in nature, two forms of vitamin K occur: vitamin K_1_ and K_2_ [61]. Several physiological processes, including bone metabolism and the regulation of specific enzyme systems, are dependent on vitamin K. Indeed, vitamin K can act as a co-factor for certain plasma proteins and play a role in immune and inflammatory responses, particularly those mediated by T cells, modulating the production of cytokines [62].

#### 3.2.2. Water-Soluble Vitamins 

The vitamins of the B group contain molecules that act as co-factors for the enzymatic reactions involved in several metabolic processes and enzymatic reactions relating to immune and growth functions [63]. It is well-established that the gut microbiota plays a role in the synthesis of a range of B vitamins, including biotin, vitamin B_12_, folates, niacin, pantothenic acid, vitamin B_6_, riboflavin and thiamin [64]. It is reported that niacin metabolites exert a significant influence on the functional characteristics of diverse leukocyte populations engaged in innate and adaptive immune responses [64]. Several studies reported that vitamin B_6_ deficiency negatively influences humoral and cell-mediated immune responses. Vitamin B_6_ acts as a co-factor in mucin metabolism, playing a key role in the synthesis of oligosaccharides and proteins [64].

Vitamin C in particular plays a significant role in immune defence by supporting the cellular functions of the innate and adaptive immune system. In particular, it has an impact on immune protection by contributing to the maintenance of the epithelial barrier against pathogens and is involved in the inflammatory response. Vitamin C modulates cytokine production and lowers histamine levels [65]. Additionally, vitamin C exhibits antioxidant capacity due to its ability to readily donate electrons, thereby protecting important biomolecules from damage caused by oxidants generated during normal cell metabolism and exposure to toxins and pollutants [39].

## 4. Vitamin Supplementation in Pigs 

It is evident that vitamin intake is crucial for maintaining optimal health, and particularly in ensuring the functionality of the immune system in pigs. Thus, it can be recommended that even in pigs, supranutritional supplementation with vitamins may be beneficial, especially during specific physiological phases. While dietary supplementation with water-soluble vitamins is not essential in pigs, as they are synthesised by the animals themselves, a reduction in synthesis has been observed in situations characterised by elevated stress levels, including the lactation and post-weaning phases [66].

It is therefore essential to ensure a regular intake of these nutrients, particularly during the most critical physiological phases when an increase in requirements is observed. In Table 2, we show the recommended vitamin requirements for sows in gestation and lactation phases and for piglets in the post-weaning phase [67].

In practice, vitamin supplementation is provided to pigs at levels exceeding the recommended requirement, given the essential role that vitamins play in maintaining health, welfare and productive performance. This is because nutritionists must integrate vitamins into the diet with a safety margin that considers the potential reduction in the vitamin’s bioavailability due to unfavourable storage conditions or time. Finally, vitamins represent a small part of the final feed cost. Therefore, significant safety margin does not have a considerable economic impact [68]. Furthermore, although there is no evidence of vitamin deficiency in conventional breeding systems, in the high-production modern genotypes, the requirement for these micronutrients may be higher than previously estimated due to the increased metabolic processes [69].

In Table 3, we report the concentrations of vitamins that usually are integrated in the diet for gestating and lactating sows and for post-weaning piglets. The dietary modification in feed vitamin concentrations is based on the scientific literature and the field experience of feed companies. It is also important to point out that nutritional recommendations of breeding companies differ according to the genetic types used, as the nutritional requirements vary greatly according to the productive performance [70]. Including appropriate levels of vitamins in pigs’ diets will not only help them reach their full genetic potential but will also improve their health, welfare and productivity.

### 4.1. Gestation and Lactation Phases

Sows go through specific physiological phases, such as gestation and lactation phases, during which supplementing the diet with a supranutritional amount of vitamins can help them cope with various stressors. It is well known that these phases require specific attention in managing and feeding. The sow must be in an adequate physical condition at farrowing, and nutrition plays a key role. In addition, during lactation the sow must have sufficient reserves not only to maintain body condition but also to support litter growth [71]. In addition, it is important to underline that vitamin integration is also essential for piglets, as they are completely dependent on the micronutrients they receive from the sow in utero and in colostrum and milk transfers. Due to the epitheliochorial nature of the porcine placenta, the transfer from sow to foetus appears to be low for fat-soluble vitamins and active for vitamin C and most water-soluble vitamins during pregnancy [72]. 

Gestation represents a crucial period during which a variety of physiological changes occur [73]. In addition, changes in the number and functionality of immune cells have been observed after 12 weeks of gestation. A decrease in the number of lymphocytes and monocytes and an increase in the number of blood neutrophils are characteristic features of the immunological profile of sows, particularly in the later stages of pregnancy [74]. Furthermore, it has been shown that prenatal stress is induced in piglets when sows are exposed to stress during pregnancy. The increased cortisol levels in sows have a negative effect on the piglets’ immune system [75]. Other sources of maternal stress can alter maternal immunity, the progress of farrowing and the quality and quantity of milk secretion. Moreover, the composition of the microbiota influences the development of the innate immune system in offspring, and this can be altered by gestational stress in sows [76].

In particular, the “transition period” is characterised by numerous physiological and metabolic changes. Understanding the physiological processes and how they change dynamically as the sow approaches farrowing, as well as colostrum production, is important because these processes have a major impact on sows’ productivity and piglets’ health, resulting in increased morbidity and mortality [77].

The many metabolic changes result in a high level of oxidative stress in sows, which becomes apparent at the end of gestation and is maintained until the end of lactation [78]. In this condition, there is an imbalance between free radical production and antioxidant defences in the body, with an excessive presence of reactive oxygen species (ROS) causing several cells and tissue alterations. In sows, this results in reduced feed intake during lactation, leading to a negative energy balance and weight loss, resulting in reduced milk production and litter weight gain [79]. Oxidative stress is caused by a marked decline in the systemic antioxidant status, characterised by a reduction in plasma vitamin E concentrations, with a negative effect on both cellular and humoral immune responses [80]. Even if dietary intake of vitamin E was high in sows, piglets may be born with low levels of vitamin E due to poor placental transfer [81]. 

Some studies have reported the effects of dietary supplementation with an increased dosage of vitamins during sows’ gestation and lactation phases on productive performance and the immune response in sows and piglets. Santos et al. [30] reported that inclusion of a dietary supplement with an extra nutritional dosage of several vitamins had no effect on the immune response of lactating piglets. Instead, other studies have shown some positive findings. Dietary supplementation with 250 IU/kg feed of vitamin E to transition sows improved IgG and IgA in sow plasma, colostrum and milk [80]. Moreover, plasma IgG, IgA levels, total antioxidant capacity and catalase activity were high in piglets from sows fed 250 IU/kg of vitamin E. Indeed, the enhancement of passive immunity and antioxidant status have a positive impact on the piglets’ growth performance. A previous study of Pinelli-Saavedra et al. [82] reported that dietary supplementation with 500 mg/kg of α-tocopheryl acetate in gestating sows significantly affected the vitamin E content of colostrum, milk, and piglet plasma without affecting immune parameters. 

Although the mechanism by which vitamin E affects immune response in pigs has not been fully elucidated, impaired lymphocyte proliferation has been reported in pigs with vitamin E deficiency [83].

Vitamin A supplementation in gestating sows enhance the immune responses after immunization with virulent rotavirus. In addition, an improvement in gut health is observed with a reduction in viral shedding and diarrhoea severity in piglets [84]. Furthermore, a recent study reported that vitamin A deficiency in pregnant sows decreased B-cell responses, reducing passive immunity, with detrimental effects on piglet mucosal immunity [85]. It has also been observed that immunisation against porcine epidemic diarrhoea virus with the supplementation of vitamin A (2645 IU/kg of feed) during the sow gestation phase improves the immune response and the passive immunity of the suckling piglets [86]. Another study showed that sows infected with porcine epidemic diarrhoea virus and fed 30,000 IU/day retinyl acetate from 76 d of gestation throughout lactation had higher colostrum and milk IgA concentrations [87].

Vitamin A deficiency in pigs impairs B- and T-lymphocyte responses and leads to an imbalance in innate and adaptive immune responses. Thus, vitamin A supplementation is crucial for the modulation of the immune system and antioxidant status in pigs [48].

It was also reported the concentration of vitamin D in milk increases in proportion to the concentration of vitamin D in the sow’s diet. A previous study of Zhang et al. [88] demonstrated how a supplementation with 0.36 mg/g calcifediol (25(OH)D_3_) in sows may have a positive impact on reproductive performance, with a shorter duration of farrowing and a higher weight of piglets at birth. Moreover, Upadhaya et al. [89] reported that 50 μg/kg 25(OH)D_3_ integrated to the basal diets of sows during gestation improved the number of piglets born alive. The average daily gain and weaning weight was also higher in piglets from sows receiving the same dosage of 25(OH)D_3_, also during lactation. 

Zhang et al. [90] reported higher levels of milk IgG in sows supplemented with 50 μg 25(OH)D_3_/kg feed. The authors also observed a higher expression of genes involved in fatty acid metabolism. They suggested that lipid metabolism is directly involved in the regulation of the immune system through the activation of macrophages. 

Vitamin C supplementation in transition sows (250, or 500 mg/kg feed) from 107 days before farrowing to 7 days after farrowing was evaluated. Even if immune parameters were not recorded, in the group fed with the highest dosage of vitamin C, a higher piglet weight at birth and at weaning was observed than in controls. No difference in piglet mortality was observed [91]. A previous study in sows (from 107 days of gestation to weaning) reported that dietary supplementation with vitamin C (2.4 g/daily) enhanced the body weight of piglets at birth and weaning and boosted the concentration of IgG and vitamin C in colostrum. It has been also observed that dietary folic acid in lactating sows (100 mg/kg feed) improved lactating piglets’ growth performances [92]. 

In Table 4, we report some studies that evaluated the effects of vitamin supplementation in sows on colostrum and plasma immune parameters.

Taken together, these data indicate that the supplementation of vitamins during gestation and lactation has a positive effect on the growth performance of sows and piglets by modulating antioxidant status and immune parameters. However, in some cases, the mechanism of action of vitamins on immune parameters is not fully elucidated.

### 4.2. Post-Weaning Phase

The post-weaning phase is one of the most critical in pig production. During this period, the animals are exposed to several stressors, including separation from the mother, a change in environment and a change in feeding habits. These factors can have a negative effect on the immature gastrointestinal system, and the occurrence of diarrhoea and enteritis has been observed, which are the result of inflammatory states [94]. In addition, post-weaning piglets are subject to a significant reduction in serum vitamin E concentrations, resulting in a reduced antioxidant status that is associated with increased susceptibility to disease [95]. As previously reported in post-weaning piglets, oxidative stress is also associated with the onset of pathologies. In fact, the antioxidant status is negatively affected by intestinal inflammation, pathogen growth and catabolic processes that weaken piglets’ health, welfare and productive performance [96]. The inflammatory response to enteric disease generates oxidative stress. This can cause tissue damage and increase the inflammatory response both locally and systemically [64]. 

Several nutritional strategies are used to improve the piglet’s health and to reduce mobility and mortality. There is evidence that the dietary supplementation of proper amounts of functional nutrients, such as vitamins, can positively affect immune parameters, lowering the risk of infection or inflammatory disease [97,98].

High-dose vitamin E supplementation (200 IU/kg feed) can positively modulate plasma vitamin E levels, which normally fall after weaning. After *E. coli* infection, an anti-inflammatory effect of high doses of vitamin E is also observed, with a significant reduction in haptoglobin levels (−34.4%) [99]. A positive effect on gut health was reported, with an increase in the ileal cell proliferation rate and tight junction protein mRNA expression in the jejunum. Amazan et al. [100] reported no effects of natural vitamin E supplementation in drinking water on serum immunoglobulin levels in piglets.

At weaning, vitamin A plays a significant role in the normal formation, development and maintenance of epithelial cells, supporting the activity of the intestinal barrier cells [101]. Moreover, Wu et al [102] reported that dietary supplementation with vitamin A (4400 IU/kg) improved antioxidant status and gut health and modified tight junction protein gene expression and the gut microbiota. The modulation of gut IL-4, IL-5 and IL-10 expression was also observed, suggesting a regulatory effect on stress-induced gut inflammation. It has also been observed that dietary supplementation with vitamin A (18,000 IU/kg of feed) reversed the adverse effects in piglets with induced diarrhoea. It reduced the incidence, intestinal immune cell infiltration and inflammatory responses [103].

Moreover, a previous study reported that encapsulated vitamin A enhances humoral immunity, with an increase in serum IgA levels [104].

Vitamin C is typically excluded from pig diets, given that pigs possess the capacity to synthesise this vitamin. However, during periods of stress, such as weaning, the levels of L-gulono-γ-lactone oxidase, an essential enzyme for vitamin C biosynthesis, may be diminished [105]. The supplementation with vitamin C in the diet of weaned piglets, due to its antimicrobial and immunomodulatory properties, would result in an enhancement of the body’s antioxidant status and, consequently, a reduction in the risk of chronic infections. Furthermore, vitamin C is capable of regenerating oxidised vitamin E, which is considered the most effective fat-soluble antioxidant with a positive modulation of humoral immune response [66]. In post-weaning piglets, dietary supplementation with vitamin C (500 mg/kg of feed) increased the humoral response [106].

It is also observed that vitamins with high antioxidant activity (A, E, C) may reduce oxidative stress induced by zerealenone [107]. A reduction of immune stimulation in piglets fed zerealenone was observed with a high dietary integration of vitamin C (150 mg/kg feed) [108].

Supplementation with vitamin D also has the potential to modulate antioxidant status, also improving immunoglobulin production. A higher dosage of vitamin D has been shown to influence enteric morphology, with an increase in villus height and crypt depth, resulting in positive effects on piglet growth. It is observed that that vitamin D supplementation (155.5 IU/kg) was associated with a reduction in the severity and duration of post-weaning diarrhoea, with a negative modulation of pro-inflammatory cytokine production [109]. The modulation of the immune system and antioxidant status in post-weaning piglets fed 25(OH)D_3_ has also been observed. Zhao et al. [110] reported that piglets fed 50 μg/kg 25(OH)D_3_ on a low Ca-P diet effectively alleviated the reduced immunoglobulin (IgG and IgA) concentrations caused by dietary Ca and P deficiency. Moreover, an improvement in weaned piglets’ immune parameters and antioxidant status has also been observed [111,112].

Dietary supplementation with folic acid (18 mg/kg feed) in post-weaning piglets improved gut health, modulating intestinal microbiota and short-chain-fatty-acid production and down-regulating the intestinal TNF-α production [81].

In Table 5, we report some studies that evaluated the effects of vitamin supplementation in piglets on serum immune and antioxidant parameters.

There is a lack of available data on the role of B vitamins in immunity in piglets. However, it is known that a deficiency of this vitamin can lead to a wide range of alterations in immune response and gut health [64]. Dietary supplementation with vitamin B_6_ (11 and 16 mg/kg) in post-weaning piglets down-regulated inflammatory cytokines and up-regulated amino acid transporters’ mRNA expression in the jejunum, without affecting other parameters [112]. It is also observed that supplementation with 20.4 mg/kg feed of vitamin B_3_ was able to increase the expression of intestinal zona occludens and decrease the diarrhoea incidence of weaned piglets [113].

These studies highlight that vitamin supplementation in piglets during the post-weaning phase has positive effect on antioxidant status, immune parameters and gut health.

## 5. Conclusions

Overall, the present data suggest that vitamins have important immunomodulatory functions in pigs. Nutritional recommendations for vitamins in pig diets do not meet the needs of modern genetic types and additional vitamins supplement appears to be crucial. In conclusion, adequate supranutritional vitamin supplementation is an effective tool for improving the immune system, antioxidant status and gut health in gestation, lactation and post-weaning phases. Further studies are needed to deepen our understanding of the role of dietary supplementation with vitamins in pigs on the immune system and gut functionality. Furthermore, the mechanisms by which vitamins can modulate the inflammatory response, gut health and microbiota in pigs are still to be elucidated.

## Figures and Tables

**Table 1:** Water-soluble vitamins and fat-soluble vitamins with their equivalent names (adapted from McDowel, 2021) [44].

Vitamins	Equivalent Names
Water-soluble	
Vitamin B_1_	Thiamin
Vitamin B_2_	Riboflavin
Vitamin B_3_	Niacin
Vitamin B_4_	Choline
Vitamin B_5_	Pantothenic acid
Vitamin B_6_	Pyridoxamine
Vitamin B_12_	Cobalamin
Vitamin C	Ascorbic Acid
Vitamin H	Biotin
Vitamin M	Folic acid
Fat-soluble	
Vitamin A_1_	Retinol
Vitamin A_2_	Dehydroretinol
Vitamin D_2_	Ergocalciferol
Vitamin D_3_	Cholecalciferol
Vitamin E	Tocopherol
Vitamin K_1_	Phylloquinone
Vitamin K_2_	Menaquinone
Vitamin K_3_	Menadione

**Table 2:** Recommended vitamin requirements for pigs in the gestation, lactation and post-weaning phases (adapted from NRC, 2012) [67].

	Estimated Requirements				
	Sow (amount/kg feed)		Piglets (amount/daily)		
	Gestation	Lactation	5–7 kg	7–11 kg	11–25 kg
Fat-soluble vitamins					
Vitamin A IU	4000	2000	585	1030	1584
Vitamin D IU	800	800	59	103	181
Vitamin E mg	44	44	4.3	7.5	10
Vitamin K mg	0.50	0.50	0.13	0.23	0.45
Water-soluble vitamins					
Riboflavin mg	3.75	3.75	1.06	1.64	2.72
Pantothenic acid mg	12	12	3.19	4.68	8.15
Niacin mg	10	10	7.98	14.05	27.16
Folacin mg	1.30	1.30	0.08	0.14	0.27
Biotin mg	0.20	0.20	0.02	0.02	0.05
Thiamine mg	1	1	0.40	0.47	0.91
Vitamin B6 mg	1	1	1.86	3.28	2.72
Vitamin B12 mg	15	15	5.32	8.20	13.58

**Table 3:** Vitamin supplements used for the gestation, lactation and post-weaning phase (adapted from Vitamin Nutrition Guidelines 2022).

	Vitamin Supplementation				
	Sow		Piglets		
	Gestation	Lactation	5 kg	5–11 kg	11–25 kg
Fat-soluble vitamins					
Vitamin A IU/kg	10,500–15,700	10,500–15,700	10,500–22,500	10,500–16,000	10,500–16,000
Vitamin D IU/kg	1570–2100	1570–2100	1890–2100	1890–2100	1890–2100
Vitamin E mg/kg	105–160	105–190	105–160	105–160	105–160
Vitamin K mg/kg	4.7–5.2	4.7–5.2	8.5–11	5.2–6.4	5.2–6.4
Water-soluble vitamins					
Riboflavin mg/kg	6.3–10.5	6.3–10.5	10.5–16	10.5–16	10.5–16
Pantothenic acid mg/kg	37–42	37–42	32–52	26–46	26–46
Niacin mg/kg	32–47	40–100	63–84	38–58	38–58
Folacin mg/kg	3.7–5.7	3.7–5.7	1.6–3.3	1.6–2.6	1.6–2.6
Biotin mg/kg	0.52–0.84	0.52–0.84	0.32–0.52	0.32–0.52	0.32–0.52
Thiamine mg/kg	2.1–2.6	2.1–3	3.8–5.8	3.2–5.2	3.2–5.2
Vitamin B6 mg/kg	3.7–5.7	3.7–5.7	6.4–8.4	6.4–8.4	6.4–8.4
Vitamin B12 mg/kg	0.032–0.052	0.032–0.052	0.052–0.072	0.042–0.062	0.042–0.062

**Table 4:** Vitamin supplements in gestating sows on colostrum and plasma immune parameters in sows and piglets.

Supplement	Dose	Animal	Effects	Treatment vs. Control, %	References
Vitamin E α-tocopheryl acetate)	250 IU/kg feed	Gestating sow from 107 d	Sow Plasma (0 d) IgG IgA α-tocopherol Piglet Plasma (21 d): IgG IgA α-tocopherol	+9.2% * +9.4% * +45.1% ** +11.4% * +9.1% * +36.3% **	[80]
Vitamin E α-tocopheryl acetate	500 mg/kg feed	Gestating sow from 0 d	Sow Plasma (103 d) α-tocopherol Colostrum: IgG Piglet serum: IgG	+84% ** NS NS	[82]
Vitamin C	2.4 g/daily	Gestating sow from 114 d	Colostrum: IgG Vitamin C	+4.5% * +28.3% **	[93]
Vitamin D 25(OH)D_3_	50 μg/kg feed	Lactating sows	Post weaning piglets Plasma: IL-6	−11% *	[89]

* for *p* < 0.05; ** for *p* < 0.01; IgG, immunoglobulin of class G; IgA, immunoglobulin of class A: IL-1, Interleuchin 1.

**Table 5:** Effect of vitamin supplementation in post-weaning piglets on immune and antioxidant parameters.

Supplement	Dose	Animal Duration	Effects	Treatment vs. Control, %	Reference
Encapsulated vitamin A	13,667 IU/kg	Weaned piglets, 42 days	Serum: GSH-Px IgA	+3.76% * +128% *	[104]
vitamin A	4400 IU/kg	Weaned piglets, 28 d	Serum: CAT IgA IgG	+28.6% * NS NS	[102]
Vitamin C	500 mg/kg	Weaned piglets, 49 d	Serum IgM	+18.9% *	[75]
Vitamin C	500 mg/kg	Weaned piglets, 68 d	Serum IgA	+36.2% *	[106]
Vitamin D 25(OH)D_3_	13,667 IU/kg	Weaned piglets, 42 d	Serum GSH-Px IgA	+53% * +131.42 *	[110]
Vitamin D 25(OH)D_3_	2000 IU/kg	Weaned piglets, 28 d	Serum IgG GSH-Px	+13.6% * +29.1% *	[111]

* for *p* < 0.05; IgG, immunoglobulin of class G; igA, immunoglobulin of class A; IgM immunoglobulin of class M; CAT, catalase; GSH-Px Glutathione Peroxidase.

## Data Availability

Not applicable.
